# Closets

**Published:** 1874-06

**Authors:** 


### CLOSETS.

CLOSETS and cellars are used by most families as receptacles for every variety of unseemly things; both are usually dark and without means of ventilation. Closets are used also for soiled clothing, which accumulates from time to time, until washing day, retaining all the peculiar affluvia, and, if any part of the clothing has been worn by a sick person, that sickness is liable to be communicated to all the members of the family. Typhoid fever, smallpox, cholera, and many other dangerous diseases have been communicated from old clothing, even months after it has been worn; the safe plan is to have every closet communicate with out-doors, so as to have a draught through every day.

